# Understanding the Societal Impact of the Social Sciences and Humanities: Remarks on Roles, Challenges, and Expectations

**DOI:** 10.3389/frma.2021.696804

**Published:** 2021-07-01

**Authors:** Benedikt Fecher, Freia Kuper, Nataliia Sokolovska, Alex Fenton, Stefan Hornbostel, Gert G. Wagner

**Affiliations:** ^1^ Research Program Knowledge and Society, Alexander von Humboldt Institute for Internet and Society, Berlin, Germany; ^2^ German Institute for Economic Research, Berlin, Germany; ^3^ Research Area Research System and Science Dynamics, German Centre for Higher Education Research and Science Studies, Berlin, Germany; ^4^ Department of Social Sciences, Humboldt University Berlin, Berlin, Germany; ^5^ Max Planck Institute for Human Development, Berlin, Germany

**Keywords:** impact, knowledge transfer, science-society interfaces, scientific advice, research utilization

## Abstract

Science is increasingly expected to help in solving complex societal problems in collaboration with societal stakeholders. However, it is often unclear under what conditions this can happen, i.e., what kind of challenges occur when science interacts with society and what kind of quality expectations prevail. This is particularly pertinent for Social Sciences and Humanities (SSH), which are part of the object they study and whose knowledge is always subject to provisionality. Here we discuss how SSH researchers can contribute to societal problems, what challenges might occur when they interact with societal stakeholders, and what quality expectations arise in these arrangements. We base our argumentation on the results of an online consultation among 125 experts in Germany (representatives from SSH, learned societies, stakeholders from different societal groups, and relevant intermediaries).

## Introduction

Societal impact is an increasingly important evaluation paradigm in science governance. This trend can be seen in the implementation of large-scale impact agendas in various research and innovation systems over the past decade. Examples include the Research Excellence Framework in the United Kingdom, the Standard Evaluation Protocol in Netherlands, or the Excellence in Research framework in Australia (van der Meulen and Rip, 2000; Geuna and Martin, 2003; Bornmann, 2013). Consequently, research is no longer assessed according to its scientific relevance alone but also according to the value it appears to generate for society. In Germany, where the present study was conducted, the societal impact of research is also at the top of the agenda of policymakers and research funders, although under a variety of terms. The German Ministry for Education and Research, for example, argues in a policy paper that a *dialogue with society* must become part of the logic of scientific reputation (BMBF, 2019).

This gradual evolution of societal impact as an evaluation paradigm was preceded by a shift in the scholarly conception of the relationship between science and society, which can be summarized as a shift “from deficit to dialogue” (Bucchi, 2008; Davies et al., 2009; Reincke et al., 2020). According to this view, science no longer provides knowledge to resolve a deficit but should develop “socially robust knowledge” together with societal stakeholders (Nowotny et al., 2001). This shift in the conception of the science-society interface implies that societal impact requires interaction between scientific and societal stakeholders. As a result, evaluation frameworks increasingly focus on processes rather than outcomes, thus rely more heavily on narratives and on formative methods more than summative ones. An example of the latter is the SIAMPI approach, which focuses on ‘productive interactions’ between science and society (Molas-Gallart and Tang, 2011; Spaapen and van Drooge, 2011).

The focus on societal impact in science governance and on interaction as a means to achieve this is particularly controversial for the social sciences and humanities (SSH), which we conceive of here as all research disciplines and subdisciplines that deal with social, societal, and cultural matters. On the one hand, from an internal scientific perspective, SSH disciplines investigate social life itself. This implies that subjects, investigators, and audiences tend to merge with one another and that value judgments might play a particularly important role (Davies et al., 2008; Cassidy, 2014). As a result, when SSH researchers interact with societal stakeholders, questions of demarcation and boundary dissolution might arise (Gieryn, 1983; Benneworth and Olmos-Peñuela, 2018). On the other hand, from an external perspective, evaluation exercises have rarely considered the particular epistemic conditions and specific utilization logics for SSH research (Reale et al., 2018). Critics have noted the mismatch between indicators and SSH notions of quality, the lack of consideration for contributions that are critical rather than solution oriented, and the overly simple framing of societal impact as economic outputs, such as the number of patents or spin-offs (Benneworth, 2015; Ochsner et al., 2017; Fecher and Hebing, 2021). Generally, established models for knowledge transfer do not do justice to the complexities of the diverse SSH disciplines and their many publics (Davies et al., 2008).

Arguably, SSH research makes important societal contributions, but these are not well understood—at least not in the governance of science. We therefore recognize a need to better understand the societal impact of SSH disciplines in terms of a) the role they might play for societal challenges, b) the problems that might arise in interactive settings that involve SSH scholars and societal stakeholders, and c) the (possibly conflicting) quality expectations that are placed on their interaction. These objectives motivate our exploratory study, which consists of an online consultation with 125 experts (i.e., SSH researchers from different disciplines along with relevant societal stakeholders). Here, we report on the results of this consultation and reflect on the implications these might have for research evaluation.

## Research Interest

### The Role of Social Sciences and Humanities Disciplines in Response to Societal Problems

There is some controversy about the role that SSH research can play in tackling societal problems: While some scholars argue that these fields should augment and emphasize their transformative potential (Sörlin, 2018; Sigurðarson, 2020), others attribute a rather passive role to them, suggesting that they should create system knowledge (i.e., knowledge that increases understanding of a social issue) or orientation knowledge (i.e., knowledge that helps to determine possibilities for action) (Becker, 2002; Jahn et al., 2012). One could furthermore argue that the public value of SSH research is not necessarily captured by their usefulness in solving problems but rather by their capacity to critically reflect on the problem itself and its potential solutions (Olmos-Peñuela et al., 2015). In this regard, the societal impact of SSH research may also be counterintuitive if one expects clear-cut solutions to problems formulated in advance. Critics of an overly narrow conception of impact as research utilization have also pointed out how social science knowledge tends to be used in diverse ways, many of which are implicit (Davies et al., 2008; Meagher et al., 2008; Stehr and Ruser, 2017). Weiss (1980), for example, observes that expertise can “creep in” as conceptual knowledge that influences ideas and decisions. Compared to the natural and technical sciences, the impact of the SSH is thought to be more indirect and less visible. While utilization of SSH might be discreet, it can also be symbolic to the extent that it is used to justify political decisions that are already made (Weiss, 1980; Albæk, 1995; Amara et al., 2004).

In summary, it is possible to identify quite different (often normative) perceptions of the societal role of SSH. Accordingly, the notion of socially relevant knowledge attributed to SSH disciplines varies: from more transformative and instrumental knowledge, to more indirect conceptual knowledge, to more counterintuitive critical knowledge. The different kinds of knowledge evoke quite different understandings of the role that the SSH should play in addressing societal challenges, which motivates our first research question (RQ1): What role is attributed to the SSH in addressing societal challenges?

### Challenges for Collaborative Arrangements Involving the SSH and Societal Actors

In the sociology of science, the shift from deficit to dialogue is associated with concepts like “Mode 2,” “post-normal science,” or “triple helix” (Funtowicz and Ravetz, 1992; Gibbons et al., 1994; Leydesdorff and Etzkowitz, 1998). These concepts all describe knowledge production as a mode of collaboration between scientific and societal stakeholders. According to a concept of transdisciplinarity, the main challenge for such collaborative arrangements is the integration of differences between actors on an epistemic, social-organizational, and communicative level (Jahn et al., 2012). As already observed above, at the epistemic level, boundaries between subjects, investigators, and audiences have a tendency to become blurred in SSH research (Davies et al., 2008; Cassidy, 2014). In collaborative arrangements that involve SSH researchers, questions of boundary work might therefore be of particular relevance (Gieryn, 1983). Furthermore, within the diverse SSH disciplines, there is little consensus on research questions and suitable methods, which poses challenges to the robustness of findings (Ochsner et al., 2017). Regarding the socio-organizational level, the structures that support societal exchange in universities are mostly centrally organized and focused on broad public communication (Peters, 2013; Marcinkowski et al., 2014; Fecher and Hebing, 2021). Questions arise as to how adequate these might be for anticipating the complexities of science in general and of the SSH in particular. Furthermore, the focus on economic indicators as a means of measuring societal impact in the past might have led to structural discrimination against SSH disciplines in organizational efforts to promote societal engagement (Benneworth and Olmos-Peñuela, 2018; Fecher and Hebing, 2021). Jacobson et al. (2004) suggest implementing an array of organizational measures that are believed to be more suitable for SSH disciplines, from increasing resources to fostering the skills of individual researchers. Regarding the communicative level, SSH researchers have frequently been accused of using overly specialized and obscure terms (Alvesson et al., 2017; Healy, 2017). At the same time, because the social sciences—and to a lesser degree, the humanities—investigate social life, they must deal with the everyday observations and *ad hoc* assumptions of the individuals with whom they engage (cf. Cassidy, 2014).

Some researchers argue that a consensus on values is not the only necessary condition for facilitating cooperation between heterogeneous actors; more importantly the conditions and structures for cooperation must be created (Star and Griesemer, 1989). For SSH disciplines, this might come with particular challenges that are not yet well understood. This motivates our second research question (RQ2): What hinders interaction between SSH researchers and societal stakeholders?

### Quality Expectations Regarding the Interaction Process

If our aim is to grasp the collaborative settings of knowledge production, we will likely need to go beyond criteria that are either purely academic or targeted towards science communication through the media (Secko et al., 2013; Rögener and Wormer, 2017). The term “socially robust,” meaning that knowledge should be scientifically robust and socially useful (Nowotny et al., 2001), is now used frequently to describe quality in these settings. Rather than bridging a cognitive gap (as purely academic projects would do), these new modes of knowledge creation aim to bridge social gaps, i.e., they are geared towards potential users, political decision makers, and entrepreneurs (Maasen and Lieven, 2006). The authors argue that in these settings, actors must develop social accountability procedures collaboratively. This undertaking produces social demands that differ from those made in disciplinary research because the researchers need to work outside the set of scientific norms that would otherwise guide their practice (Merton, 1973; Mitroff, 1974). This creates new requirements vis-à-vis the outcome. These outcomes are not easily located on a disciplinary map but instead suit the context of application (Gibbons et al., 1994). This will most likely be accompanied by processual requirements to bridge the above-mentioned gaps and to deal with the specific contexts that are addressed by these arrangements.

There are general preconceptions about how collaborative modes of knowledge production might consolidate the quality conceptions of all parties involved. Still, these often remain at an abstract level, which motivates our third research question (RQ3): What do scientific and societal stakeholders perceive as the conditions for good interaction?

## Data and Methods

The study is exploratory in that it aims to better understand the societal impact of SSH disciplines by an empirical examination of the role ascribed to SSH research in addressing societal challenges, as well the quality expectations arising in collaborative processes involving SSH researchers. Our findings are based on an online consultation of SSH researchers, societal stakeholders, and intermediaries. We subsequently discussed the results of the consultation with SSH and science researchers in two workshops, where we further scrutinized their implications for assessing the societal impact of SSH.

The selection of participants in the consultation process was deliberate and targeted a) researchers from different SSH disciplines who had experience of knowledge transfer and b) societal stakeholders from politics, media, business, culture, civil society, and public administration who had experience in collaborating with SSH scholars. In order to ascertain that participants did indeed have experience of collaboration, we conducted preliminary interviews, researched specific collaboration projects, and, in the case of researchers, asked learned societies for nominations. The deliberate selection of participants was necessary in order to ensure that respondents could legitimately provide answers to the partly normative questions. Our final sample consists of 125 responses, of which 36 are SSH scholars, 71 societal stakeholders, and 18 intermediaries. Of the SSH scholars, four participants came from core humanities disciplines (philosophy, legal studies, history), four from economics, thirteen from other social sciences, and one each from pedagogy, linguistics, and design research. Twelve of the researchers did not indicate their disciplinary background. Further, our sample includes a group we describe as “intermediaries.” These are individuals that are involved in managing and enabling collaborations between SSH researchers, for example communications officers at universities or independent science communication consultants. We chose to include this group in the consultation because we assumed that they would be uniquely positioned to observe and thus reflect on the conditions of these interactions. Table 1 illustrates the final expert sample by group membership.

**TABLE 1:** Sample of the online consultation by group membership.

Participants	No.
**SSH scholars**	**36**
In universities	27
In nonuniversity research institutes	9
**Societal stakeholders**	**71**
NGOs	19
Politics	10
Public administration	11
Private sector	11
Cultural sector	1
Media	19
**Intermediaries**	**18**

The consultation consisted of an online survey that comprised both a close-ended section on sociodemographics and a set of mainly open-ended questions about individual experience in collaborative settings involving SSH researchers. Our analysis of the three research questions is based on five open questions in the survey (Table 2). One of the questions refers to the Covid-19 pandemic (Table 2; RQ1). We chose to include this because the pandemic is a complex societal challenge and is thus relevant to the subject of the study.

**TABLE 2:** Research interest and survey questions.

Research interest	Survey question
Role of SSH researchers (RQ1)	From your perspective: For which societal issues are the SSH research particularly relevant?
	How do you assess the role played by SSH disciplines in solving societal problems, for instance during the Covid-19 pandemic
Interaction challenges (RQ2)	Where have you experienced problems and challenges in communicating and applying the results of SSH research?
	How would you assess the role of scientific institutions (universities, non-university research institutions)? Where do you recognize concrete potential for development in the relationship between science and society in these institutions?
Quality expectations (RQ3)	Please describe what constitutes good collaboration or exchange between science and society. If possible, please also address what special requirements apply to the SSH.

We conducted a structuring content analysis in order to analyze the textual data. This technique corresponds to the inductive technique of qualitative content analysis (Mayring, 2000) and takes into account Kuckartz’s structuring method by using an interpretative initial processing to then iteratively form consistent categories (Kuckartz, 2014). Quotations in this paper are the authors’ translations from the original German responses into English.

We encouraged the experts to publish their names and responses because we consider them relevant for further research: 103 agreed to publish their responses, 68 agreed to publish their names and institutions, 27 to publish only the name of their institutions, and 30 wished to stay anonymous. The survey instrument, the anonymized MAXQDA file, as well as the full answers of those who granted permission, can be found on the project website.

This study had limitations regarding the selection of participants in the consultation: Despite every effort being made to recruit a diverse and relevant set of participants, the selection can hardly reflect the diversity of SSH researchers and its many specialized societal stakeholders. Further research is necessary to understand the manifestations of the generic categories presented here in different contexts.

## Results

From the survey responses, we first identify topics that SSH research is associated with and the role SSH research fulfills within society. Second, we present the challenges that are mentioned when SSH researchers and societal stakeholders interact. Third, we turn to quality expectations in this interaction. In each results section, we will report on the findings by referring to the number of codes ascribed to a category in brackets and use exemplary quotes where suitable.

### Role of Social Sciences and Humanities Researchers

From the responses regarding the societal issues that SSH expertise is relevant for, we were able to identify 31 societal issues that span nearly every aspect of social and natural life, as well as technical innovation. Broadly, these can be assigned to the following categories: “politics” (45), “economy” (47), “culture” (6), “education” (26), “ecology” (56), “civil society” (131), “health” (34), and “technology” (42).

The answers likely relate to the respondents’ particular interests and expertise and do not represent those areas of real-world problems that the SSH contribute to. However, the issues show that the spectrum of topics ascribed to SSH disciplines goes far beyond narrow disciplinary couplings (e.g., educational research that deals with education or economics that deal with economic growth) and includes contemporary and frequently transformative topics, such as climate change, migration, or the current pandemic. The ubiquity of potential issues for SSH engagement is expressed in this quote from a journalist:

“Every topic has a societal component—from fundamental questions of democracy and politics to questions concerning nature and technology. Basically, each question that requires social action and regulation” (Media_ID103, 10).

While these issues provide some indication of the wide topical range for potential SSH engagement, the participants’ perception of the role of SSH research in addressing these societal issues might provide a more accurate picture of how that engagement might actually unfold. We coded the answers to the question of how participants assess the role of SSH research in solving societal problems accordingly. In total, we identified six distinct societal roles that are frequently referred to by the experts: explaining, reflecting, educating, signaling, foresight, and informing (Table 3).

**TABLE 3:** Societal functions of SSH knowledge.

Roles	Description	Example	#Codes
Explain	To describe and contextualize an issue.	“It is always about identifying—understanding—explaining and providing contextual knowledge. That is always of importance” (Economy_ID132, 10).	77
Reflect	To discuss and interpret an issue.	“What does it mean that one part of the population can work from home in a relatively safe manner, while another part of the population cannot, and is thus potentially more exposed?” (NGO_ID85, 25).	65
Educate	To build competence in a specific area.	“[SSH] should develop intercultural competences” (Media_ID180, 15).	7
Signal	To point to an issue.	“Impulses for necessary discourses can and should also come from [SSH] research” (NGO_ID200, 10).	20
Foresee	To predict the development of an issue.	“The potential implications of current research have societal relevance—technological developments such as CRISPR Cas 9 or AI should be discussed more widely in society so that we can negotiate ethical issues raised by the introduction of such technologies early enough” (Intermediary_ID174, 10).	21
Inform	To support decision-making.	“Solid analyses of socio-political developments, numerical data, and impact assessments are needed in politics and administration. They are picked up on and incorporated into decision-making” (PublicAdmin_ID61, 16).	80

We found indications that each of these six functions correspond to different types of knowledge. For example, the “explain” category relates to system knowledge needed to understand a social issue because it contains statements from participants that are geared towards contextualizing social issues without suggesting any concrete instructions for action. By the same token, the “educate” category contains knowledge used to build competence in a specific issue area. The category “foresee” relates to knowledge needed to determine possibilities for decision-making as it contains statements from participants that refer to future developments. For example, one person working in public administration describes SSH research as an “early warning system for problems that have not yet become apparent” (PublicAdmin_ID61, 9). According to this statement, SSH disciplines should assess the societal implications of social change. These include, as several respondents state, the implications of artificial intelligence on the future of work.

The “inform” category is closely linked to what is referred to as the instrumental use of SSH knowledge, i.e., it is used directly for decision-making. Both the “reflect” and the “signal” categories resonate with what might be considered critical knowledge. Statements in the “reflect” category do not refer to the provision of expertise for problem solving but to interpreting and analyzing the problem and the solution. The “signal” category includes statements that, according to the participants in the consultation, refer to issues that receive too little attention but are considered relevant to public discourse or policymaking. Accordingly, the role of SSH disciplines is to point to these problematic aspects and to act as a critical observer. In relation to the Covid-19 pandemic, for example, the participants mentioned that SSH researchers emphasized the psychological, social, and cultural consequences of pandemic control. Some experts believe SSH expertise is not given enough attention in current political strategies, others like this intermediary describe their influence as lagged but present:

“Whereas at the beginning it was mainly the virologists who were heard, in my opinion the social sciences have now made themselves heard in many respects and have pointed out numerous important aspects of economic and socio-political relevance. For example, the fact that the daycare centers and schools have not yet been closed again is not only due to the virological assessment that children are less likely to spread the virus, but also due to the indications of the problems for working parents and for the children whose educational disadvantages have been exacerbated” (Intermediary_ID110, 24).

The statements from politicians in our sample frequently referred to the “foresee” category, but other than that there were no striking quantitative variations in the distribution of codes.

With regard to the roles attributed to the SSH in solving societal problems, we identified different levels of activity, from a rather passive, contextualizing role (e.g., “explain”) to a more active, influencing role (e.g., “inform”). This leads us to conclude that the SSH provide a diverse range of problem-relevant kinds of knowledge for societal challenges. From a solution-focused point of view, SSH knowledge is partly counterintuitive because it does not necessarily aim to contribute to a solution but seeks to question the problem and its solution. Moreover, rather than producing knowledge that might itself stimulate change or even transformation, SSH disciplines are more frequently attributed the role of producing “cohesion knowledge,” that is, knowledge that helps anticipate change. In this regard, SSH research fulfils a moderating role in complex change processes by helping to establish and maintain social order, cohesion, and equality. In our view, the multiple roles attributed to SSH disciplines could amount to a moderating role that would involve taking into account the complexity of issue formation in change processes as well as attempts to tackle these. Therefore, SSH disciplines are in a position to consider overarching issues of social cohesion and equality. The capacity of SSH research to address questions of cohesion is strongly reflected in the frequency of references to issues: the terms equality or inequality are mentioned 79 times by the respondents, democracy is mentioned 32 times, and cohesion or similar terms are mentioned 28 times.

### Interaction Challenges

In order to understand where difficulties arise in the interaction between SSH scholars and societal stakeholders, the participants were asked about the problems and challenges they experienced in previous interactions and—in order to assess organizational aspects—the role of universities in supporting science-society interactions. We identified four kinds of interaction challenges in the answers: 1) translational challenges that relate to different modes and logics of interaction, 2) institutional challenges that relate to the governance and organization of science, 3) epistemic challenges that relate to knowledge creation processes of SSH disciplines, and 4) uptake challenges that relate to the use of SSH expertise by different societal stakeholders. Table 4 presents these challenges and their subdimensions.

**TABLE 4:** Interaction challenges.

Challenges	Categories	Example	#Codes
Translational challenges	Language barriers	“Challenges in applying the results of social science research also lie in the different ways in which journalists and scientists work with language” (SSHscholar_ID178, 14).	27
	Conflicting system logics	“Politics has to make decisions and win majorities or, create acceptance. Science can give recommendations, but this might just result in different recommendations coexisting [...]” (Intermediary_ID110, 12).	73
Institutional challenges	Lack of resources	“The everyday routine at the university, with extensive teaching and exams obligations and increasingly also administrative tasks, which coincides with shrinking resources, already leaves little room for research. This means that the Third Mission is an additional burden” (SSHscholar_ID192, 12).	19
	Lack of organizational support	“Institutions should create structured incentive systems for scientists to raise awareness of societal challenges and to consider what they themselves can contribute to solving them” (SSHscholar_ID65, 28)	19
	Lack of rewards	“The transfer (not only the publication) of research results should be valued as an important aspect of scientific work in education but also in evaluations” (Intermediary_ID195, 13).	20
Epistemic challenges	Ambiguity of results	“But in contrast to the natural sciences, there are rarely any clear “truths” here. So it’s not easy for the media to present a comprehensive and well-balanced picture when selecting scientific contributions” (PublicAdmin_ID61, 25).	23
	Conflicting paradigms	“One challenge is the question of how issues that are scientifically controversial can be presented to the public in such a way that the reputation of science does not suffer and, ideally, this heterogeneity can even be used productively” (SSHscholar_ID179, 13).	9
Uptake challenges	Lacking appreciation of SSH expertise	“I see challenges in the general perception and appreciation of social science research being too low” (Intermediary_ID201, 13).	27
	Public attention dynamics	“Provocation is better “received” than factuality; “loud” colleagues are simply more seen and heard” (SSHscholar_ID67, 13).	13
	Risk of instrumentalization	“Politics must not misuse scientific findings for its own agendas and thereby partly discredit them” (Economy_ID96, 18).	5

### Translational Challenges: Conflicting System Logics and Boundary Work

Translational challenges relate to different modes and logics of interaction between involved parties. The category comprises statements made by respondents that refer to semantic aspects and systemic differences between science and other social systems that hamper meaningful interaction. The statements in this category can be split into two categories: “language barriers” (27) and “conflicting system logics” (73).

Some participants perceive the *language* of SSH scholars to be complicated, as this journalist describes:

“As a journalist, it strikes me that social science researchers very often and unfortunately quite naturally use terms that are hardly used or understood by the general public” (Media_ID159, 13).

Differences, however, can be found in the assessment of language barriers. Some see the use of technical concepts as a necessity for describing social phenomena in a differentiated way, while others see it as unnecessarily complicated prose that is a hindrance to productive exchange. In general, references to language barriers are mostly made by participants working in the private sector or in the media.

A second challenge can be described as “conflicting system logics.” Statements in this category refer to three closely related aspects of incompatibility: 1) temporality of SSH research (i.e., SSH research takes time and cannot satisfy needs immediately), 2) conflicting notions of relevance (i.e., societal relevance of SSH is not based on immediate societal needs), and 3) self-referentiality of SSH research (i.e., SSH research refers to itself and not to what others consider social problems). The conflicting system logics resulting from these are well expressed in a quote from an SSH researcher, who on the one hand calls for SSH researchers to anticipate different societal contexts (here the media) but on the other hand reports that this can lead to conflicts among academic peers:

“Scholars should recognize that they move in a different system logic when they communicate with the media, for example. I experience a lot of criticism of the portrayal of science in the media, which I consider inappropriate. Of course, there is a decrease in length, but that is also completely okay.” (SSHscholar_ID138, 16–17)

In general, the participants often refer to different system logics, usually to explain why an exchange could not take place from their specific perspectives. In this quote, for example, a politician reports on the context of his decision making and the associated lack of time to deal with SSH research:

“Science is a different system than politics; there is a democracy proviso; being an elected official does not give me enough time to read or receive scientific literature.” (Politics_ID196, 17)

Different system logics explain the translational challenges between SSH researchers and members of other social subsystems, specifically with regards to language usage, the notions of relevance, and time and content-related use considerations. This explanation can be problematic when functional differentiation of social systems is used as a pretext for not engaging in interaction at all. It might be more fruitful to think of the interaction between societal stakeholders and scientists as one where boundaries between science and nonscience are contextually and continuously dissolved and redrawn.

### Institutional Challenges: Mismatch Between Aspiration and Resources

Institutional challenges relate to the governance and organization of science. In this respect, we identified three types of challenges in the statements. These are “lack of resources” (19), “lack of organizational support” (19), and “lack of rewards” (20).

In most cases, references to lack of resources refer to limits concerning SSH researchers’ time and skills. One social scientist mentioned the need for training for research staff when explaining the latter:

“[We] are not trained to do this; we usually do basic research and teach basic science at universities—we need knowledge transfer” (SSHscholar_ID68, 15).

A second institutional challenge relates to the lack of organizational support. Respondents often refer to a decoupling of transfer infrastructures at universities and the researchers working there, or to necessary investment in transfer capacities at research organizations. The latter becomes clear in this statement made by a participant who works in public administration:

“In my opinion, scientific institutions should invest more in public relations—these positions are often sparsely staffed and funded [...]. The relevance of the job/intermediary function is recognized more and more, but this is (often) not yet reflected in the structures” (Intermediary_ID229, 13).

A third challenge in this category is the lack of rewards for societal engagement, which the participants link to the academic reputation and funding system. Another social scientist describes what she perceives as an undervaluation of engagement as follows:

“[There is a] lack of reputation for this activity as opposed to third-party funding and high-ranking publications. [Engagement] is only an “add on”” (SSHscholar_ID44, 13).

The notion of “engagement as an add-on” (i.e., not a main task) is mentioned frequently and especially by SSH scholars in the consultation. However, the participants discuss the matter of recognition with significant differentiation: One expert describes societal impact as an additional pathway for scholarly work, *alongside* scientific impact:

“Since publication excellence can hardly be mitigated, they could instead create funding lines that can only be used if the relevance to the SDGs is laid out clearly,” (SSHscholar_ID65, 28).

Lack of recognition for public engagement activities and a lack of resources to carry them out are not specific to SSH disciplines per se. However, they may be more pronounced here because knowledge transfer is even less rewarded and incentivized in a dominant framework focused on economic outcomes. If strengthening societal engagement is a science policy priority, the results here suggest that there is a perceived mismatch between this aspiration and the resources allocated to it.

### Epistemic Challenges: The Illusion of Stable Social Sciences and Humanities Knowledge

The epistemic challenges category describes challenges that relate to the knowledge creation of SSH disciplines. It includes two subcategories, “ambiguous results” (23) and “conflicting paradigms” (9).

With respect to “ambiguous results,” statements often contain comparisons to the “hard” natural sciences, where results are perceived by some participants to be clear and unambiguous. In contrast, results from SSH disciplines are often described as vague. For example, for a respondent who works as a researcher and in the media, this is the main reason why results from the natural sciences are preferred:

“Questions and research designs are often too vague, the results too ambiguous. Therefore, journalists prefer communicating results from the natural sciences” (SSHscholar_ID142, 16).

The “conflicting paradigms” category contains statements that emphasize how different schools of thought within SSH disciplines result in different ways of understanding and assessing the same issue. A social scientist in the consultation interpreted the heterogeneity of SSH disciplines as an impediment to communication:

“Distinctive disciplinarity and families of methods in SSH disciplines prevent common problem-oriented communication” (SSHscholar_ID206, 22).

While the heterogeneity of SSH disciplines is often described as normal and indeed as an asset by the participants, some point to a problem, namely that this lack of consensus can also be perceived by the public as a lack of scientific rigor. This can lead to a loss of reputation and trust.

“One challenge is the question of how issues that are scientifically controversial can be presented to the public in such a way that the reputation of science does not suffer and, ideally, this heterogeneity can even be used productively” (SSHscholar_ID179, 13).

Of course, conflicting paradigms and ambiguous results are not purely SSH problems. However, they manifest in specific ways there. In general, SSH disciplines comprise very different approaches, research questions, and epistemological premises. Moreover, their results are often strongly dependent on context. These characteristics are echoed in our respondents’ view of the ambiguity of SSH results, which they describe as a challenge when interacting with societal stakeholders.

### Uptake Challenges: Lacking Appreciation and Public Attention Dynamics

The category uptake challenges includes statements from participants that relate to the use of SSH expertise by societal stakeholders. We identified three types of uptake challenges. These are: “lacking public appreciation” (27), “public attention dynamics” (13), and the “risk of instrumentalization” (5).

Regarding “lacking appreciation,” SSH disciplines are, again, often contrasted with the natural sciences by participants. Many of them describe the natural sciences as having a comparatively higher public status, which becomes obvious in this statement from an SSH scholar:

“From my point of view, we offer many research topics that are of interest to a broader public, but we are not yet perceived and treated equally with the natural sciences” (Intermediary_ID229, 16)

This observation is backed up by a journalist who explains that while disciplines such as medicine, physics, or engineering are met with fascination, SSH disciplines are not:

“While the natural sciences and medicine are often met with widespread fascination for their subjects in society, this is often lacking in social science. Physics and technology are sexy, other disciplines are not” (Media_ID159, 16).

The “dynamics of public attention” subcategory subsumes statements that describe SSH research as being out of kilter with the public interest. In general, this refers to a perceived mismatch between the utilitarian perspective of societal stakeholders and the supply of knowledge that SSH disciplines can provide. Often, participants refer to the fast pace of social media, which SSH research cannot keep up with. Some participants even describe adverse effects when SSH researchers adapt their communication to the dynamics of publicity, which is made obvious in a quote from a humanities scholar, who explains how attention might trump relevance in public communication:

“Provocation is better “received” than factuality; “loud” colleagues are simply better seen and heard” (SSHscholar_ID67, 13).

The “risk of instrumentalization” category is rarely referenced. We list it nevertheless, because it is often mentioned in the literature and is distinct from the other listed challenges. The category subsumes statements that refer to the misuse of SSH expertise for political interests. For instance, a representative working in the economy and for an NGO states:

“Politicians must not misuse scientific findings for their own agendas and thereby partly discredit them” (Economy_ID96, 18).

Taken together, when SSH results are discussed by the public, they appear to not be appreciated in the same way as natural science results. Instead. they are made subject to attention dynamics and might be instrumentalized. This negative perception might be linked to the subtle nature and multiple ways in which SSH expertise reaches the public and political decision makers. If media attention factors determine whether SSH results are noted by the public, the scientific and societal relevance of SSH expertise might recede.

### Quality Expectations

The third research question addresses quality expectations, i.e., conditions for a good exchange between societal stakeholders and SSH researchers. To this end, we asked the participants open questions about their expectations for a good exchange and about the specific conditions that might apply to SSH disciplines. From the answers, we are able to identify eight distinctive quality expectations that can be divided into three main categories. These are 1) process-related, b) outcome-related, and c) person-related quality expectations (Table 5). Engagement with society, albeit an aspiration of many research organizations, seems to be difficult in current organizational structures according to our respondents.

**TABLE 5:** Quality expectations.

Quality expectation	Categories	Example	#Codes
Process	Comprehensibility	“Summarize findings in a generally understandable, audience-oriented, and brief and concise manner” (NGO_ID60, 16).	26
	Form	“Knowledge should be transferred to the public through various and adapted transfer formats and communication channels, for example, transfer forums, workshops, lecture series as formats that can be used in a way that is appropriate to the target group and audience” (Intermediary_ID108, 16).	25
	Inclusivity	“Co-creative exchange between science and non-scientific actors is important. Each group contributes specific knowledge needed for complex problem solving” (SSHscholar_ID232, 15).	26
	Pertinence	“Knowledge and presumption must be clearly separated in the dialogue with society” (Economy_ID163, 36–37).	13
Outcome	Transparency	“It seems important to me that science communication also openly names the weaknesses of science. For example, peer review is no guarantee of quality” (SSHscholar_ID138, 30).	30
	Relevance	“At the same time, the relevance of science to the reality of life must be recognizable and tangible. This last point in particular is often missing in the social sciences” (Media_ID57, 20).	31
Person	Empathy	“Good cooperation means engaging with the other side and listening without prejudice” (SSHscholar_ID224, 16).	67
	Disinterestedness	“In my view, a good exchange is characterized above all by the fact that it is not primarily guided and inspired by the self-promotional intentions of individual scientists or scientific organizations” (SSHscholar_ID37, 16).	14

### Process-Related Quality Expectations

Process-related quality expectations refer to the interaction between SSH scholars and societal stakeholders and includes the codes “comprehensibility” (26), “pertinence” (13), “inclusivity” (26), and “form” (25).

“Comprehensibility” encompasses statements that refer to the mutual understanding between actors. Typically, these statements refer to comprehensible and clear communication of results on the part of SSH scholars and the adaptation to interlocutors. Accordingly, complex contents should be conveyed in such a way that those involved in the dialogue are able to follow and respond in an informed manner. The code “pertinence” refers to statements that suggest that knowledge should be used in a problem—and solution-oriented manner. This is illustrated by a statement made by a politician:

“For the policy sphere, I would like to see more focused exchanges that bring in key research findings” (Politics_ID237, 19).

“Inclusivity” refers to the actors involved in an interaction. We distinguished between two types of inclusivity. The first is selective inclusivity, which means that appointed experts who can contribute relevant and specific expertise should be involved. The second is universal inclusivity, which implies broader participation involving those who are possibly affected by the issue. Some participants point out that diverse expertise is needed to achieve viable results. Lastly, statements coded as “form” typically refer to the existence of an interaction format that is adequate for exchange and problem-solving.

It is impossible to meet all of these expectations of the interaction process. One SSH scholar puts it in these almost utopian terms:

“The goal should be to communicate complexity, reflexivity, and provisionality simply, clearly, understandably, and plausibly” (SSHscholar_ID205, 15).

It can be assumed that the more complex a problem is and the more diverse the parties involved in the interaction process, the more difficult it will be to arrive at some form of shared meaning. In this regard, there are expected tensions between inclusivity, pertinence, and comprehensibility, while formality might imply a strategy to meet these expectations in the best possible way.

### Outcome-Related Quality Expectations

Outcome-related quality expectations refer to the results of an interaction process between SSH scholars and societal stakeholders. This category comprises the codes “transparency” (30) and “relevance” (31).

The code “transparency” indicates statements that refer to two kinds of transparency: 1) method transparency and 2) motivation transparency. In this article, we use method transparency to refer exclusively to SSH disciplines and signal the requirement of communicating uncertainties and clearly describing methods as necessary for good exchange. Motivation transparency refers to the communication of motivating factors (e.g., personal interest, dependencies, client expectations) and pertains to both SSH scholars and societal stakeholders. This is made obvious in a statement from a social science scholar:

“As part of society, scientists perceive and research socially relevant topics—politics should make the use of scientific research results transparent” (SSHscholar_ID68, 18).

“Relevance” includes statements that refer to the practical implications of the interaction process. We distinguished between individual and societal relevance. Individual relevance signifies the benefits for the individuals involved and is described by some as a motivating factor for partaking in the interaction process. Societal relevance is usually viewed in a differentiated way as referring either to benefits for individual citizens or benefits for specific groups and sectors of society. In some statements, such as the following made by a politician, societal relevance is framed as a return on societal investment in publicly financed research:

“Society makes a considerable contribution to the financial security and freedom of science, not least through public budgets. It can therefore expect science to take an interest in societal issues and to make its contribution to solving societal problems [...]” (Politics_ID234, 18).

However, achieving both transparency and relevance might be difficult, as this statement from an economics scholar shows:

“The greatest challenge in communicating social science research is often to openly acknowledge the uncertainty inherent in its findings while convincing people that they nevertheless contain important information” (SSHscholar_ID157, 16).

In this case, transparency is seen as a hindrance for relevance. Further tensions might arise when personal and societal relevance do not correspond, or when transparency (in the sense of replicability) cannot be achieved. There might also be a conflict between different quality expectations in the outcome of the interaction process.

### Person-Related Quality Expectations

Person-related quality expectations refer to the individuals involved in the interaction process. They subsume the codes “empathy” (67) and “disinterestedness” (14).

“Empathy” indicates statements that refer to the mutual acknowledgement of all parties involved. Most statements in this category refer to acknowledging the position of the other parties involved in the interaction process. Typically, the social position of an individual comes with certain concessions, for example, journalists are granted reporting duties, politicians have decision-making power, and SSH scholars possess research autonomy. The reciprocal nature of the expectation of empathy is made clear in this quote from a journalist in the consultation:

“When researchers recognize that the media are their partners—in discourse, in presentation, in criticism. That means being available for media inquiries, discussing issues of relevance with a journalist, and sharing material. It also means tolerating exaggerations, even if one’s own business is differentiation” (Media_ID114, 16).

Some participants state that empathy should not be blind but informed. This is made obvious in a quote from a participant who works in public administration:

“It is important that the results of SSH disciplines can be properly assessed. Excessive claims in the social sciences, in the sense of objective truths, can easily produce disappointment and lead to a deviation, which in the worst cases can then leave the impression of arbitrariness of the decisions and actions under discussion” (PublicAdmin_ID167, 15).

The code “disinterestedness” is used for statements that emphasize that actors should not pursue their own interests but act for the benefit of society. This is often combined with the expectation that personal opinions should be separated from facts and that the conversation should be devoid of emotions and self-promotional intentions. Responding to the question of what constitutes a good collaboration between science and society, one SSH scholar states:

“In my view, a good exchange is characterized above all by the fact that it is not primarily guided and inspired by the self-promotional intentions of individual scientists or scientific organizations” (SSHscholar_ID37, 16).

There are conflicts between disinterestedness and empathy, for instance when it comes to the proclaimed necessity of leaving emotions aside. In addition, there may be potential cross-category tensions between person—and outcome-related quality expectations, for instance in relation to disinterestedness and the individual relevance described above. The same holds true for informed empathy and inclusivity. Remarkably all participants, researchers as well as societal stakeholders from different fields, name the quality expectation empathy most frequently as a condition for exchange. Reflection on ones own position seems crucial for science-society-interactions.

## Discussion

In this article, we used an expert consultation to examine the societal impact of SSH disciplines, i.e., the role of SSH research in addressing societal issues, as well as the resulting challenges and quality expectations. The results shed light on the conundrum of addressing societal issues while being part of the subject matter.

### Social Sciences and Humanities Knowledge as Cohesion Knowledge

The societal issues that SSH disciplines relate to are broad and transcend disciplinary couplings. The quasi ubiquity of SSH impact areas resonates with recent research findings (e.g., Bastow et al., 2014). The roles ascribed to SSH disciplines in addressing societal problems are likewise diverse and range from more instrumental tasks, such as informing a policy decision, to more contextualizing activities, such as explaining the social implications of a problem. The latter resonates with Stehr and Ruser’s (2017) description of social scientists as “meaning producers,” i.e., their knowledge does not focus on practical choices but on processes of meaning, which may give rise to decisions. In addition, we find evidence of a more counterintuitive role for SSH disciplines in addressing societal challenges, namely critiquing the definition of a problem and the envisaged solution. This finding resonates with Burchell (2009) who proposes that, from a societal perspective, the social sciences might best be interpreted as a “critical friend” (see also Davies et al., 2008). Participants in the consultation describe the relevance of this critical capacity, for instance, in discussing the social, cultural, and psychological implications of the Covid-19 pandemic, which some feel have not been sufficiently considered in policy decisions.

Along with these roles, we identified different types of knowledge that SSH disciplines can provide to help resolve societal challenges. These range from overview and system knowledge, as described by Becker (2002), to instrumental knowledge (Fähnrich and Lü; Stehr and Ruser, 2017) like the kind that is used to inform political decision-making processes. This differentiation resonates with (Weiss, 1980) who suggests that the contributions of SSH research to decision-making processes are much wider than a narrow idea of knowledge utilization suggests. Moreover, “critical knowledge,” i.e., knowledge that enables us to question societal decisions, appears to be an essential contribution of SSH disciplines to societal issues. This positions SSH researchers as a critical corrective in addition to its contextualizing and co-creating capacity. At a higher level of abstraction, we observe that SSH disciplines are rarely associated with “transformative knowledge” that causes change (Becker, 2002) but instead with knowledge that helps us anticipate societal transformations and to deal with change (see also Sigurðarson, 2020). We refer to this kind of knowledge as “cohesion knowledge.”

### Continuous Boundary Work

In the scholarly debate, dialogue between representatives from both science and society is understood as a condition for “socially robust” knowledge, i.e., knowledge that is both scientifically robust and socially useful (Nowotny et al., 2001). Consequently, we conceptualize interaction as a prerequisite for societal impact (see also Spaapen and van Drooge, 2011). This motivated us to interrogate challenges in interactive and problem-oriented settings involving SSH disciplines. The challenges we identify can be categorized as translational, institutional, epistemic, and uptake challenges, and they thus correspond roughly to the framework suggested by Jahn et al. (2012). While many of the challenges we identified point to contingent issues, some results stand out.

When it comes to translation, reducing linguistic complexity without being accused of triviality and commonplace hypotheses is a core challenge for SSH disciplines. Some of the societal stakeholders in the consultation describe SSH disciplines as self-referential and the language used as unnecessarily complicated at times. Bridging the “social gap” (Maasen and Lieven 2006) between science and society thus means that SSH scholars must adapt their language (e.g., their use of terms), although at the risk of compromising their epistemic authority. A problem-oriented interaction with societal stakeholders, however, might contribute to increased “methodological efficiency” as a form of continuous external validation (Woolgar, 2000). Regarding institutional challenges, we find initial evidence for a structural disadvantage of SSH disciplines. This might be explained with reference to the fact that the established entrepreneurial heuristic of societal impact carries little significance for SSH disciplines (Benneworth and Olmos-Peñuela, 2018). Epistemic challenges mostly concern the heterogeneity of SSH disciplines and their approaches, intermittently conflicting paradigms, and the dynamic object of study, i.e., society as a moving target (Dayé, 2014). It follows that SSH disciplines produce knowledge that is highly context-dependent, situated, and dynamic (Gattone, 2012; Fähnrich and Lüthje, 2017). Hence, there are serious limitations regarding the extent to which objective, stable, and context-independent knowledge can be expected from SSH disciplines (Davies et al., 2008). This finding is consistent with the self-conception of many SSH disciplines as critical, reflective, and contextual. When it comes to the uptake of SSH knowledge, the consulted representatives note how SSH expertise is not always fully appreciated and may explain to a certain extent the lack of appreciation for SSH research. For example, in the consultation, SSH research is often contrasted with natural science and technical disciplines, whose results are not only perceived as more stable but often as more exciting, too. This resonates with Knudsen (2017), who found a deficit framing for the humanities in Danish print media. Cassidy (2014) explains this lack of appreciation with the close relationship of SSH disciplines to everyday life: “Unlike most natural sciences, where the specialist training, knowledge and equipment of scientists grants them largely uncontested expertise, social scientists’ expertise is often about matters of everyday experience and common-sense knowledge” (p. 190).

Taken together, these challenges suggest a twofold implication: The calls for more resources and recognition are on the one hand contingent issues that can give impulses to the governance of science. On the other hand, our results illustrate how the position of the SSH in society is a matter of ongoing negotiations. The identified challenges show how the SSH are caught up in boundary work in their interactions with extra-academic fields (Gieryn, 1983). They speak of troubles of SSH researchers to claim their authority, which is linked to epistemic dynamics, that find expression in language usage, specific temporalities and context-specific results. How the SSH position themselves towards their moving target, the society, becomes even more of a challenge in collaborative formats.

### Contextual Quality Configurations

Our empirical findings indicate a three-dimensional framework for ensuring quality in collaborative arrangements involving SSH researchers and societal stakeholders. The first is process-related and describes the expectations of the exchange itself. The second is person-related and describes the expectations towards the people involved. The third is outcome-oriented and includes the expectations of the outcome. In collaborative settings, there will most likely be contradictory expectations of what entitles persons to participate, how interacting partners should behave, and what constitutes relevant knowledge (see also Kropp and Wagner, 2010). This leads to conflicts between different expectations of quality that are difficult to avoid, for instance between disinterestedness and empathy, but also within categories, for instance, regarding different understandings of relevance (e.g., how can scientific demands for relevance be reconciled with demands for utility?). At times, the participants in the consultation offer solutions to these conflicts between quality expectations, for instance when they say that there are conditions for participation in the interaction such as having a basic understanding of the other interaction partner. This is in line with Bromme’s (2020) concept of “informed trust,” according to which it needs not only trust in public scientific statements but also knowledge on the system of science to make an informed judgement. Our findings add a nuance to this hypothesis by suggesting that informed trust must be reciprocal, i.e., researchers participating in a dialogue must also understand the societal stakeholders they engage with.

Generally, we can safely assume that the more diverse and complex the setting for a dialogue is, the more difficult it may be to document expertise and to establish transparency. If being affected by an issue legitimizes participation in a dialogue, then it may be more difficult to enforce pertinence as a premise. If expertise legitimizes participation, there is also a risk of exceeding the level of fact. It follows that there must be legitimate reasons for trade-offs between different quality expectations. These should depend on the aim of the interaction, the individuals involved, and the chosen interaction format. It follows that quality expectations in collaborative settings should not be understood universally, unilaterally, and statically. Instead, they should be considered within their specific context, reciprocally, and dynamically. Hence, we propose that quality itself must be an object of these interactions, i.e., there should ideally be deliberation about the appropriate quality configuration for the problem at hand. This could be particularly relevant for SSH disciplines, which, as discussed above, have to engage in continuous boundary work due to their position in society. The outline of a quality framework as proposed here can be a basis for deliberating on the quality of these arrangements. That said, for particularly established forms of interaction (e.g., scientific policy advice), there may already be recognized default settings from which it is possible to extrapolate.

## Conclusion

Our results show, that the societal impact of SSH disciplines can be counterintuitive and precisely *not* aimed at solving a problem. Instead, they often seek to challenge both the problem and its solution. Nor does SSH research necessarily strive for transformation but instead seeks an understanding and a moderation of social change. Therefore, the impact of the SSH is often discreet, indirect, and conceptual. Thus, the quality of the societal impact of SSH disciplines can only be understood in relation to their specific context, in the sense that it is person-, problem-, and time-dependent and must take into account different field logics as it takes place in a “space between fields” (Williams, 2020). For these reasons, a rigid, purely quantitative assessment of societal impact of SSH disciplines should generally be avoided, especially with regard to how assessment shapes and stabilizes underlying values (Espeland and Sauder, 2007; Williams, 2020).

Our results provide some arguments for so-called formative evaluations of the societal impact of SSH disciplines. Formative evaluations focus on the process (e.g., an interaction, a program, or a project) while the activities are ongoing. They are geared towards learning and goal adjustment. The SIAMPI approach (Spaapen and van Drooge, 2011) as well as the Agora model (Frederiksen et al., 2003; Barré, 2010) or Public Value Mapping (Bozeman and Sarewitz, 2011) are promising examples of such formative assessment concepts. Using the concept of “productive interactions,” the SIAMPI approach focuses on the individual’s contributions to an interaction rather than reactively assessing its outputs. With its emphasis on productivity however, it cannot capture the counterintuitive contributions outlined above, which do not focus on the solution to a problem but instead question the problem.

Nonetheless, this at times counterintuitive impact of SSH disciplines may not be suitable for evaluation at all. Instead, it might imply that additional measures such as capacity building are needed to support the interaction between science and society (Sigurðarson, 2020). The integration of science communication, and with it the reflection on boundaries, must become an integral part of science education. This is underlined by the trend towards public legitimation of research funds and a new social contract for science not as hasty obedience to a political desire but as a basis for an informed discussion of perspectives and implications. In that sense, it seems reasonable to reflect on and gain a more nuanced understanding of the societal impact of SSH disciplines within research communities and learned societies.

## Data Availability

The raw data supporting the conclusions of this article will be made available by the authors, without undue reservation.

## References

[B1] Albæk E. (1995). Between Knowledge and Power: Utilization of Social Science in Public Policy Making. Policy Sci 28, 79–100. 10.1007/BF01000821

[B2] Alvesson M. Gabriel Y. Paulsen R. (2017). Return to Meaning: A Social Science with Something to Say. First edition. Oxford: Oxford University Press. 10.1093/oso/9780198787099.001.0001

[B3] Amara N. Ouimet M. Landry R. (2004). New Evidence on Instrumental, Conceptual, and Symbolic Utilization of University Research in Government Agencies. Sci. Commun. 26, 75–106. 10.1177/1075547004267491

[B4] Barré R. (2010). Towards Socially Robust S&T Indicators: Indicators as Debatable Devices, Enabling Collective Learning. Res. Eval. 19, 227–231. 10.3152/095820210X512069

[B5] Bastow S. Dunleavy P. Tinkler J. (2014). The Impact of the Social Sciences: How Academics and Their Research Make a Difference. Los Angeles; London; New Delhi; Singapore. Washington, D.C: SAGE. 10.4135/9781473921511

[B6] Becker E. (2002). Transformations of Social and Ecological Issues into Transdisciplinary Research. Knowl. Sustain. Dev. Insight Encycl. Life Support. Syst. 3, 949–963.

[B7] Benneworth P. Olmos-Peñuela J. (2018). Reflecting on the Tensions of Research Utilization: Understanding the Coupling of Academic and User Knowledge. Sci. Public Pol. 45, 764–774. 10.1093/scipol/scy021

[B8] Benneworth P. (2015). Tracing How Arts and Humanities Research Translates, Circulates and Consolidates in society. How Have Scholars Been Reacting to Diverse Impact and Public Value agendas?How Have Scholars Been Reacting to Diverse Impact and Public Value Agendas? Arts Humanities Higher Edu. 14, 45–60. 10.1177/1474022214533888

[B9] BMBF (2019). Grundsatzpapier des Bundesministeriums für Bildung und Forschung zur Wissenschaftskommunikation. Berlin: Federal Ministry of Education and Research.

[B10] Bornmann L. (2013). What Is Societal Impact of Research and How Can it Be Assessed? a Literature Survey. J. Am. Soc. Inf. Sci. Tec 64, 217–233. 10.1002/asi.22803

[B11] Bozeman B. Sarewitz D. (2011). Public Value Mapping and Science Policy Evaluation. Minerva 49, 1–23. 10.1007/s11024-011-9161-7

[B12] Bromme R. (2020). “Informiertes Vertrauen: Eine psychologische Perspektive auf Vertrauen in Wissenschaft,” in Wissenschaftsreflexion. Editors Jungert M. Frewer A. Mayr E. (Brill | mentis), 105–134. 10.30965/9783957437372_006

[B13] Bucchi M. (2008). “Of Deficits, Deviations and Dialogues. Theories of Public Communication of Science,” in Handbook of Public Communication of Science and Technology. Editors Bucchi M. Trench B. (London; New York: Routledge), 57–76. Available at: http://site.ebrary.com/id/10236333 (Accessed April 13, 2021).

[B14] Burchell K. (2009). A Helping Hand or a Servant Discipline? Sci. Technol. Innov. Stud. 5, 49–61. 10.17877/DE290R-970

[B15] Cassidy A. (2014). “Communicating the Social Sciences: a Specific Challenge?,” in In Routledge Handbook of Public Communication of Science and Technology. Editors Bucchi M. Trench B. (London; New York: Routledge, Taylor & Francis Group), 186–197.

[B16] Davies H. Nutley S. Walter I. (2008). Why 'knowledge Transfer' Is Misconceived for Applied Social Research. J. Health Serv. Res. Pol. 13, 188–190. 10.1258/jhsrp.2008.008055 18573770

[B17] Davies S. McCallie E. Simonsson E. Lehr J. L. Duensing S. (2009). Discussing Dialogue: Perspectives on the Value of Science Dialogue Events that Do Not Inform Policy. Public Underst. Sci. 18, 338–353. 10.1177/0963662507079760

[B18] Dayé C. (2014). Visions of a Field. Sci. Technol. Hum. Values 39, 877–891. 10.1177/0162243914538323

[B59] Espeland W. N. Sauder M. (2007). Rankings and Reactivity: How Public Measures Recreate Social Worlds. Am. J. Sociolo. 113, 1–40. 10.1086/517897

[B20] Fähnrich B. Lüthje C. (2017). Roles of Social Scientists in Crisis Media Reporting: The Case of the German Populist Radical Right Movement PEGIDA. Sci. Commun. 39, 415–442. 10.1177/1075547017715472

[B21] Fecher B. Hebing M. (2021). How Do Researchers Achieve Societal Impact? Results of an Empirical Survey Among Researchers in Germany. Berlin: SSOAR Available at: https://www.ssoar.info/ssoar/handle/document/71327.

[B22] Frederiksen L. F. Hansson F. Wenneberg S. B. (2003). The Agora and the Role of Research Evaluation. Evaluation 9, 149–172. 10.1177/1356389003009002003

[B23] Funtowicz S. O. Ravetz R. (1992). “Three Types of Risk Assessment and the Emergence of post-normal Science,” in Social Theories of Risk (Praeger), 251–274.

[B24] Gattone C. F. (2012). The Social Scientist as Public Intellectual in an Age of Mass Media. Int. J. Polit. Cult. Soc. 25, 175–186. 10.1007/s10767-012-9128-1

[B25] Geuna A. Martin B. R. (2003). University Research Evaluation and Funding: An International Comparison. Minerva 41, 277–304. 10.1023/B:MINE.0000005155.70870.bd

[B40] Gibbons M. Limoges C. Nowotny H. Schartzmann S. Scott P. B. Trow M. (1994). in The New Production of Knowledge. The Dynamics of Science and Research in Contemporary Societies. London (Thousand Oaks, Calif: SAGE Publications). 10.4135/9781446221853

[B26] Gieryn T. F. (1983). Boundary-Work and the Demarcation of Science from Non-science: Strains and Interests in Professional Ideologies of Scientists. Am. Sociological Rev. 48, 781. 10.2307/2095325

[B27] Healy K. (2017). Fuck Nuance. Sociological Theor. 35, 118–127. 10.1177/0735275117709046

[B28] Jacobson N. Butterill D. Goering P. (2004). Organizational Factors that Influence University-Based Researchers' Engagement in Knowledge Transfer Activities. Sci. Commun. 25, 246–259. 10.1177/1075547003262038

[B29] Jahn T. Bergmann M. Keil F. (2012). Transdisciplinarity: Between Mainstreaming and Marginalization. Ecol. Econ. 79, 1–10. 10.1016/j.ecolecon.2012.04.017

[B30] Knudsen S. (2017). Thinking inside the Frame: A Framing Analysis of the Humanities in Danish Print News media. Public Underst. Sci. 26, 908–924. 10.1177/0963662517693452 29025368

[B31] Kropp C. Wagner J. (2010). Knowledge on Stage: Scientific Policy Advice. Sci. Technol. Hum. Values 35, 812–838. 10.1177/0162243909357912

[B32] Kuckartz U. (2014). Qualitative Text Analysis: A Guide to Methods, Practice & Using Software. Los Angeles: SAGE Publications.

[B33] Leydesdorff L. Etzkowitz H. (1998). The Triple Helix as a Model for Innovation Studies. Sci. Public Pol. 25, 195–203. 10.1093/spp/25.3.195

[B34] Maasen S. Lieven O. (2006). Transdisciplinarity: a New Mode of Governing Science? Sci. Public Pol. 33, 399–410. 10.3152/147154306781778803

[B35] Marcinkowski F. Kohring M. Fürst S. Friedrichsmeier A. (2014). Organizational Influence on Scientists' Efforts to Go Public. Sci. Commun. 36, 56–80. 10.1177/1075547013494022

[B36] Mayring P. (2000). Qualitative Content Analysis. Forum Qual. Sozialforschung Forum Qual. Soc. Res. 1 Available at: http://www.qualitative-research.net/index.php/fqs/article/view/1089. (Accessed August 20, 2015).

[B37] Meagher L. Lyall C. Nutley S. (2008). Flows of Knowledge, Expertise and Influence: a Method for Assessing Policy and Practice Impacts from Social Science Research. Res. Eval. 17, 163–173. 10.3152/095820208X331720

[B38] Merton R. K. (1973). “The Normative Structure of Science,” in The Sociology of Science: Theoretical and Empirical Incvestigations (Chicago: Univ. of Chicago Pr), 267–278.

[B41] Mitroff I. I. (1974). Norms and Counter-norms in a Select Group of the Apollo Moon Scientists: A Case Study of the Ambivalence of Scientists. Am. Sociological Rev. 39, 579. 10.2307/2094423

[B42] Molas-Gallart J. Tang P. (2011). Tracing “productive Interactions” to Identify Social Impacts: an Example from the Social Sciences. Res. Eval. 20, 219–226. 10.3152/095820211X12941371876706

[B60] Nowotny H. Scott P. B. Gibbons M. (Editors) (2001). Re-Thinking Science. Knowledge and the Public in an Age of Uncertainty. London: Sage Publications.

[B43] Ochsner M. Hug S. Galleron I. (2017). The Future of Research Assessment in the Humanities: Bottom-Up Assessment Procedures. Palgrave Commun. 3, 17020. 10.1057/palcomms.2017.20

[B44] Olmos-Peñuela J. Benneworth P. Castro-Martínez E. (2015). Are Sciences Essential and Humanities Elective? Disentangling Competing Claims for Humanities' Research Public Value. Arts Humanities Higher Edu. 14, 61–78. 10.1177/1474022214534081

[B45] Peters H. P. (2013). Gap between Science and media Revisited: Scientists as Public Communicators. Proc. Natl. Acad. Sci. 110, 14102–14109. 10.1073/pnas.1212745110 23940312 PMC3752168

[B46] Reale E. Avramov D. Canhial K. Donovan C. Flecha R. Holm P. (2018). A Review of Literature on Evaluating the Scientific, Social and Political Impact of Social Sciences and Humanities Research. Res. Eval. 27, 298–308. 10.1093/reseval/rvx025

[B47] Reincke C. M. Bredenoord A. L. van Mil M. H. (2020). From Deficit to Dialogue in Science Communication. EMBO Rep. 21, E51278. 10.15252/embr.202051278 32748995 PMC7506985

[B48] Rögener W. Wormer H. (2017). Defining Criteria for Good Environmental Journalism and Testing Their Applicability: An Environmental News Review as a First Step to More Evidence Based Environmental Science Reporting. Public Underst. Sci. 26, 418–433. 10.1177/0963662515597195 26265708

[B49] Secko D. M. Amend E. Friday T. (2013). Four Models of Science Journalism. Journalism Pract. 7, 62–80. 10.1080/17512786.2012.691351

[B50] Sigurðarson E. S. (2020). Capacities, Capabilities, and the Societal Impact of the Humanities. Res. Eval. 29, 71–76. 10.1093/reseval/rvz031

[B51] Sörlin S. (2018). Humanities of Transformation: From Crisis and Critique towards the Emerging Integrative Humanities. Res. Eval. 27, 287–297. 10.1093/reseval/rvx030

[B52] Spaapen J. van Drooge L. (2011). Introducing “Productive Interactions” in Social Impact Assessment. Res. Eval. 20, 211–218. 10.3152/095820211X12941371876742

[B53] Star S. L. Griesemer J. R. (1989). Institutional Ecology, `Translations' and Boundary Objects: Amateurs and Professionals in Berkeley’s Museum of Vertebrate Zoology, 1907-39. Soc. Stud. Sci. 19, 387–420. 10.1177/030631289019003001

[B54] Stehr N. Ruser A. (2017). Social Scientists as Technicians, Advisors and Meaning Producers. Innovation: Eur. J. Soc. Sci. Res. 30, 24–35. 10.1080/13511610.2016.1207505

[B55] van der Meulen B. Rip A. (2000). Evaluation of Societal Quality of Public Sector Research in the Netherlands. Res. Eval. 9, 11–25. 10.3152/147154400781777449

[B56] Weiss C. H. (1980). Knowledge Creep and Decision Accretion. Knowledge 1, 381–404. 10.1177/107554708000100303

[B57] Williams K. (2020). Playing the fields: Theorizing Research Impact and its Assessment. Res. Eval. 29, 191–202. 10.1093/reseval/rvaa001

[B58] Woolgar S. (2000). Social Basis of Interactive Social Science. Sci. Pub. Pol. 27, 165–173. 10.3152/147154300781782039

